# *Leucophyllum frutescens* mediated synthesis of silver and gold nanoparticles for catalytic dye degradation

**DOI:** 10.3389/fchem.2022.932416

**Published:** 2022-09-29

**Authors:** Bansuri Gami, Khalida Bloch, Shahansha M. Mohammed, Srikanta Karmakar, Satyajit Shukla, Adersh Asok, Sirikanjana Thongmee, Sougata Ghosh

**Affiliations:** ^1^ Department of Microbiology, School of Science, RK University, Rajkot, India; ^2^ Functional Materials Section (FMS), Materials Science and Technology Division (MSTD), CSIR-National Institute for Interdisciplinary Science and Technology (NIIST), Thiruvananthapuram, India; ^3^ Department of Polymer Science and Technology, Calcutta University, Kolkata, India; ^4^ Academy of Scientific and Innovative Research (AcSIR), Ghaziabad, India; ^5^ Photosciences and Photonics Section, Chemical Sciences and Technology Division (CSTD), CSIR-National Institute for Interdisciplinary Science and Technology (NIIST), Thiruvananthapuram, India; ^6^ Department of Physics, Faculty of Science, Kasetsart University, Bangkok, Thailand

**Keywords:** *Leucophyllum frutescens*, silver nanoparticles, gold nanoparticles, optimization, rhodamine B dye, photocatalysis

## Abstract

The application of nanotechnology is gaining worldwide attention due to attractive physico-chemical and opto-electronic properties of nanoparticles that can be also employed for catalytic dye degradation. This study reports a phytogenic approach for fabrication of silver (AgNPs) and gold nanoparticles (AuNPs) using *Leucophyllum frutescens* (Berl.) I. M. Johnst (Scrophulariaceae) leaf extract (LFLE). Development of intense dark brown and purple color indicated the synthesis of AgNPs and AuNPs, respectively. Further characterization using UV-visible spectroscopy revealed sharp peak at 460 nm and 540 nm for AgNPs and AuNPs, respectively that were associated to their surface plasmon resonance. High resolution transmission electron microscope (HRTEM) revealed the spherical shape of the AgNPs, whereas anisotropic AuNPs were spherical, triangular and blunt ended hexagons. The majority of the spherical AgNPs and AuNPs were ∼50 ± 15 nm and ∼22 ± 20 nm, respectively. Various reaction parameters such as, metal salt concentration, temperature and concentration of the leaf extract were optimized. Maximum synthesis of AgNPs was obtained when 5 mM for AgNO_3_ reacted with 10% LFLE for 48 h at 50°C. Likewise, AuNPs synthesis was highest when 2 mM HAuCl_4_ reacted with 10% LFLE for 5 h at 30°C. Energy dispersive spectroscopy (EDS) showed phase purity of both the nanoparticles and confirmed elemental silver and gold in AgNPs and AuNPs, respectively. The average hydrodynamic particles size of AgNPs was 34.8 nm while AuNPs was 140.8 nm as revealed using dynamic light scattering (DLS) that might be due to agglomeration of smaller nanoparticles into larger clusters. ZETA potential of AgNPs and AuNPs were 0.67 mV and 5.70 mV, respectively. X-ray diffraction (XRD) analysis confirmed the crystallinity of the nanoparticles. Fourier transform infrared spectroscopy (FTIR) confirmed that various functional groups from the phytochemicals present in LFLE played a significant role in reduction and stabilization during the biogenic synthesis of the nanoparticles. The bioreduced AgNPs and AuNPs catalytically degraded Rhodamine B dye (RhB) in presence of UV-light with degradation rate constants of 0.0231 s^−1^ and 0.00831 s^−1^, respectively. RhB degradation followed a first order rate kinetics with 23.1 % and 31.7% degradation by AgNPs and AuNPs, respectively.

## Introduction

Nanotechnology has received wide attention globally owing to the attractive surface properties of the nanoparticles that range between 1–100 nm in size (Cheriyamundath and Vavilala, 2021). Physical, chemical, optical and electronic properties of the nanomaterials are largely dependent on their size and shape (Dauthal and Mukhopadhyay, 2016). Large scale application of nanotechnology include almost every aspect of life such as, textiles, paints, sensors, electrical equipments, agriculture, and even therapeutics (Jadoun et al., 2021). Smaller dimension and large surface area makes the nanoparticles ideal for functionalization with drugs, contrast agents and targeting ligands that is significant for biomedical applications (Aftab et al., 2018; Karmakar et al., 2021). Nanoparticles can be fabricated employing top-down and bottom-up approaches. Conventional physical methods for synthesis of metal nanoparticles include lithography, laser ablation, milling and sputtering. Likewise, chemical methods for nanoparticle synthesis includes hydrothermal, sol-gel, pyrolysis and vapour deposition (Bloch et al., 2021). However, these methods involve hazardous reaction conditions and toxic chemicals during synthesis for reduction of the metal ions to respective nanoparticles and their stabilization thereafter (Tarannum and Gautam, 2019). Moreover, energy consumption, high cost and requirement of sophisticated instruments are major limitations in these methods. Hence, more recently, biogenic metal and metal oxide nanoparticles are reported from bacteria, algae, fungi, medicinal plants and their metabolites with diverse therapeutic applications (Ali et al., 2020; Ghosh and Webster, 2021).

Medicinal plant mediated synthesis of nanoparticles is more advantageous as the plant extracts used in the synthesis process are nontoxic and environmentally benign. The green synthesis method is simple, rapid, economical, and perform under moderate operational conditions with low energy consumption (Ahmed and Mustafa, 2020). Various plant parts such as root, stem, bark, leaf, flower, fruit, and even latex are reported for synthesis of nanoparticles that is preferred over microbe-mediated synthesis as this process neither require stringent aseptic condition, nor long incubation periods (Chandra et al., 2020; Hano and Abbasi, 2022). Secondary metabolites of medicinal plants are superior reducing and stabilizing agents that facilitate the synthesis of nanoparticles with multiple applications (Khatami et al., 2018). Rich phytochemical diversity that include predominance of terpenoids, flavones, ketones, aldehydes, alkaloids and amides play a significant role in the nanoparticle synthesis (Gour and Jain, 2019). Among various medicinal plants *Leucophyllum frutescens* (Berl.) I.M. Johnst (Scrophulariaceae) is a drought tolerant shrub that can sustain even in unfavourable summer heat (Mohammed and Al-Megrin, 2021). Diterpenoid leubethanol from *L. frutescens* was reported to have potent antimicrobial activity against multi-drug-resistant tuberculosis (Miller et al., 2020). Anthocyanin, carotenoids, lutein, lycopene, and phenolics in *L. frutescens* not only render it with the antimicrobial property but also rationalize its traditional use in treatment of asthma, cataracts, cough, dysentery, and liver injury (Menchaca et al., 2013). Hence, it would be interesting to explore the nanobiotechnological potential of *L. frutescens*.

In recent years, the excessive use of synthetic and organic dyes in textiles, tannery, cosmetics and food industries has posed a severe threat to the environment. Various conventional methods employed for the removal of toxic synthetic dyes from water bodies are often insufficient and ineffective. Nanoparticles exhibit tremendous catalytic potential that can be exploited for dye degradation for effective treatment of the industrial effluents. Various phytogenic nanoparticles such as, silver, gold, copper, zinc, platinum, palladium, etc. with photocatalytic effect are reported for disinfection, and water treatment by catalytic dye degradation (Iravani, 2011; Khan et al., 2018). Dyes are toxic colouring agents that are largely used in textile, food, paper, and pharmaceutical industries and are released in huge amount in the environment (Naghizadeh et al., 2021). Consumption of the dye contaminated water has deleterious effect on the health due to oxidative stress mediated damage and carcinogenesis.

Among various synthetic dyes, Rhodamine B (RhB) is a water soluble xanthene dye that is widely used as a trace dye for the determination of rate and direction of water flow in industries. Hence this dye is considered as one of the most common organic pollutants in the environment (Awad et al., 2021). Hereby, photodegradation of dye pollutants to less harmful products is an effective remedial method to control their adverse ecological impact. Several conventional treatment methods like chemical oxidation, sonochemical degradation, microwave, photoelectric, and solar photofenton are often inadequate, expensive and complicated (Baruah et al., 2018). Therefore, development of more facile and eco-friendly approach for toxic dye removal is a prerequisite for clean environment. Hence, use of biogenic nanoparticles for catalytic dye degradation can serve as a promising alternative strategy for controlling the environmental pollution (Ismail et al., 2018).

This novel study is the first report where critical reaction parameters like duration, metal precursor concentration, temperature and concentration of plant extracts are thoroughly optimized for maximum green synthesis of silver (AgNPs) and gold (AuNPs) nanoparticles using *L. frutescens* leaf extract (LFLE). The resulting AgNPs and AuNPs were characterized using advanced analytical techniques like high resolution transmission electron microscopy, energy dispersive spectroscopy, dynamic light scattering and X-ray diffraction spectroscopy. This is the first report on photocatalytic RhB dye degradation by phytogenic AgNPs and AuNPs synthesized using LFLE.

## Materials and methods

### Plant extract preparation

Preparation of *L. frutescens* leaf extract (LFLE) was carried out as per our earlier report (Ranpariya et al., 2021). In short, *L. frutescens* leaves were collected from RK. University, Rajkot, Gujarat, India in the month of February. The plant was authenticated by taxonomist at the Saurashtra University, Rajkot, India with the voucher specimen number BG001. Mature and fresh leaves from the plant were thoroughly washed in running tap water for 15 min and then shade dried for 3 days at room temperature. Dried leaves were pulverized into fine powder using an electric blender. Thereafter, 5 g of the leaf powder was suspended in 100 ml of distilled water in a 300 ml Erlenmeyer flask followed by boiling for 20 min. The extract obtained was filtered through Whatman filter paper No. 1 and the filtrate was collected and stored at 4°C for further experiments.

### Synthesis of AgNPs and AuNPs

Reduction of Ag^+^ ions was initiated on addition of 1 ml of LFLE to 9 ml of 5 mM aqueous silver nitrate (AR grade) solution. Thereafter, the flasks were incubated in darkness under shaking condition (120 rpm) at 40°C (Soshnikova et al., 2018). During the synthesis of AuNPs, reduction of Au^3+^ ions started immediately on addition of 1 ml of LFLE to 9 ml of 1 mM aqueous chloroauric acid (AR grade) solution followed by which the flasks were incubated under aforementioned conditions (Alaqad and Saleh, 2016). Progress in the synthesis of both the nanoparticles was monitored by recording the UV-visible spectra of the reaction mixture at regular intervals using an UV-1900 Shimadzu spectrophotometer.

### Optimization study

Different reaction parameters like metal salt concentration, temperature, and LFLE concentration were optimized. Temperature optimization for the synthesis of AgNPs and AuNPs was carried out at different temperature ranging from 4°C to 50°C. In order to optimize concentration of metal salt for the synthesis of AgNPs and AuNPs, the concentration of AgNO_3_ and HAuCl_4_ were varied respectively, from 0.3 mM to 5 mM. The effect of variation in LFLE concentration from 1% to 10% was also checked. Progress of the reactions were monitored using UV-visible spectrophotometry at regular intervals (Shinde et al., 2018). Statistical significance was determined by analysis of variance (ANOVA two factor) with *p* < 0.05.

### Characterization of synthesized AgNPs and AuNPs

Morphological analysis of the phytogenic AgNPs and AuNPs was accomplished in a high resolution transmission electron microscope (HRTEM) Tecnai G2 F30 operating at an accelerating voltage of 300 kV by placing a droplet of sonicated sample directly on a carbon-coated copper grid that was subsequently dried under infrared (IR) lamp for 20 min. Energy dispersive spectroscopy (EDS) were obtained using an energy dispersive spectrometer at 0–20 keV energy range to confirm the elemental composition. The hydrodynamic size and zeta potential of the AgNPs and AuNPs were measured in polystyrene cuvettes employing dynamic light scattering (DLS) Microtrac system. In short the freshly synthesized samples were sonicated for 30 min to remove any agglomeration and poured in the cuvettes followed by particle size analysis and zeta potential measurement. A thin film of concentrated nanoparticles was prepared on a thoroughly washed grease-free clean glass slide and desiccated overnight to obtain a moisture free layer. The phase and crystallographic structure of synthesized AgNPs and AuNPs was determined by subjecting to thin film X-ray diffraction (XRD) using an X-ray wavelength of 1.5406 Å, current and voltage settings of 40 mA and 40 kV, respectively, with a 2θ ranging from 20° to 90°.

### Fourier transmission infrared spectroscopy

The alteration of the functional groups in LFLE before and after synthesis of AgNPs and AuNPs were analyzed using FTIR. After 5 h of synthesis of the nanoparticles the reaction mixture was centrifuged at 10,000 rpm for 15 min at room temperature. The pellet was redispersed in sterile distilled water and the supernatant was collected. The process of centrifugation and redispersion in sterile distilled water was repeated three times to ensure better separation of free entities from the nanoparticles. The purified pellet was then dried and subjected to FTIR measurement using the potassium bromide (KBr) pellet technique. The nanoparticle powder was mixed with KBr (AR grade) and exposed to an infrared source of 600–4000 cm^−1^ for identification of the functional groups associated with the nanoparticles. Likewise, the functional groups in the recovered supernatant after the reaction was also compared with the crude unreacted LFLE.

### Photocatalytic dye degradation

The procedure used for the measurement of photocatalytic activity of samples is similar to the one described earlier elsewhere (Zachariah et al., 2008). A 0.4 g L^−1^ of sample was added to 125 ml of 7.5 μM RhB (Practical grade) dye solution in distilled water having an initial pH of 6.67. The resulting suspension was equilibrated by stirring in darkness (that is, without the UV irradiation) for 1 h to stabilize the adsorption of RhB on the sample surface. The aqueous suspension was then subjected to the UV irradiation in the photoreactor chamber (LZC-4X, Luzchem, Canada) by using the 14 UVA lamps (6 top and 8 side lamps) having an emission peak intensity of 350 nm with the continuous in-built magnetic stirring for 1 h. An 8 ml aliquot was frequently taken out at 10 min interval followed by centrifugation (Hettich EBA 20, Sigma-Aldrich labware, Bengaluru, India). The filtrate was examined by using a UV-visible absorption spectrophotometer (UV-2401 PC, Shimadzu, Japan) to determine the residual RhB concentration in the aqueous dye solution (Baiju et al., 2007).

$$RhB_{adsorbed}\left(\%\right)=\frac{C_{-60}-C_{0}}{C_{-60}}\times 100=\frac{A_{-60}-A_{0}}{A_{-60}}\times 100$$ (1)

where, *C*_−*60*_ and *C*_*0*_ are the RhB concentrations within the aqueous solution before (time (*t*) = −60 min) and after (*t* = 0 min) the adsorption experiment conducted in the dark condition; while, *A*_−*60*_ and *A*_*0*_ are the corresponding absorbance values. The normalized concentration of RhB remaining in the solution after stirring in the dark condition for 1 h is calculated by using Eq. 2.

$$RhB_{residual}\left(\%\right)=\frac{C_{0}}{C_{-60}}\times 100=\frac{A_{0}}{A_{-60}}\times 100$$ (2)

The normalized concentration of RhB remaining in the solution under the UV irradiation is calculated by using Eq. 3.

$$RhB_{residual}\left(\%\right)=\frac{C_{t}}{C_{-60}}\times 100=\frac{A_{t}}{A_{-60}}\times 100$$ (3)

where, *C*_*t*_ is the RhB concentration remaining within the aqueous dye solution after the UV irradiation time of *t* = *t* min; while, *A*_*t*_ is the corresponding absorbance value.

The first order kinetic constant (k) for the degradation of RhB is calculated using Eq. 4

$$\ln\frac{C_{0}}{C_{t}}=kt$$ (4)

## Results and discussion

### UV-visible spectroscopy and optimization studies

**FIGURE 1:**
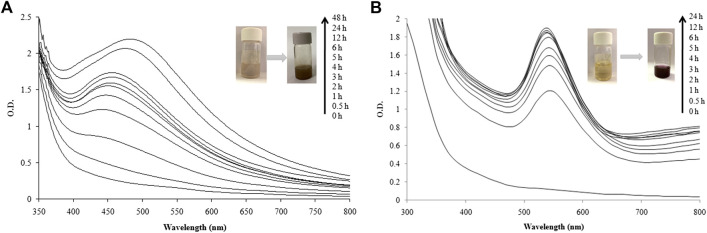
UV-visible spectra recorded as a function of reaction time for nanoparticle synthesis using LFLE. **(A)** AgNPs; and **(B)** AuNPs.

The green synthesis of nanoparticles is an attractive efficient route for synthesis of metal and metal oxide nanoparticles. UV-visible spectra of the reaction mixture of LFLE and AgNO_3_ were recorded at regular time intervals. Reduction of Ag^+^ to AgNPs by LFLE could be followed by color change from yellow to dark brown as shown in the inset of Figure 1A. Although there was no significant synthesis at t = 0 h and t = 0.5 h, the synthesis of AgNPs was initiated after 1 h followed to which there was steady increase in the intensity of peak at 460 nm up to 48 h. Likewise, reduction of Au^3+^ to AuNPs by LFLE could be followed by color change initially from yellow to light purple that turned further dark as observed in the inset of Figure 1B. Synthesis of AuNPs was extremely faster and was found to initiate immediately at t = 0.5 h. Subsequent rise in peak at 540 nm was noted till 5 h of synthesis followed to which no further increase in the peak insentity was noticed. Appearance of the brown and purple colour indicating AgNPs and AuNPs were found to be identical with the earlier reports with *Sisymbrium irio* and *Trachyspermum ammi* extracts, respectively (Mickymaray, 2019; Perveen et al., 2021).

These findings rationalize LFLE as a promising reducing and stabilizing agent that successfully reduced Ag^+^ and Au^3+^ ions into AgNPs and AuNPs, respectively within 5 h. The synthesis was carried out at ambient reaction conditions unlike conventional methods such as chemical reduction, sol-gel method, and/or photochemical method (Lkhagvajav et al., 2011; Gabriel et al., 2017; Ranoszek-Soliwoda et al., 2017). No external chemical reducing or capping agents were required as the phytochemicals in the LFLE satisfied the purpose hence making this one-pot synthesis more economical unlike the physical and chemical methods (Bonigala et al., 2018). Our results supporting the nanobiotechnological potential of *L. frutescens* is in agreement with the earlier reports on synthesis of nanoparticles from medicinal plants such as, *Cleome viscosa* (Lakshmanan et al., 2018), *Carica papaya* (Banala et al., 2015), *Phytolacca decandra, Hydrastis canadensis, Thuja occidentalis* (Das et al., 2013), and *Amomum villosum* (Soshnikova et al., 2018).

Phytogenic synthesis of metal nanoparticles depends on several parameters such as reaction time, concentration of metal salts, temperature, and plant extract concentration (Ahmed and Mustafa, 2020). The syntheses of both AgNPs and AuNPs by LFLE were completed at 5 h which was faster as compared to the reported synthesis of AgNPs by *Pimpinella anisum* seed extract that took 96 h (Alsalhi et al., 2016). Likewise, *Withania somnifera* extract was able to synthesize AgNPs only after 7 days which substantiate the fact that LFLE mediated nanoparticles synthesis is rapid which is advantageous over others (Marslin et al., 2015). It is speculated that the reaction time is a key factor determining the shape, size and stability of nanoparticles. Size of nanoparticles increases with time and hence shorter reaction time is beneficial to have smaller nanoparticles with larger surface area (Alsalhi et al., 2016).

**FIGURE 2:**
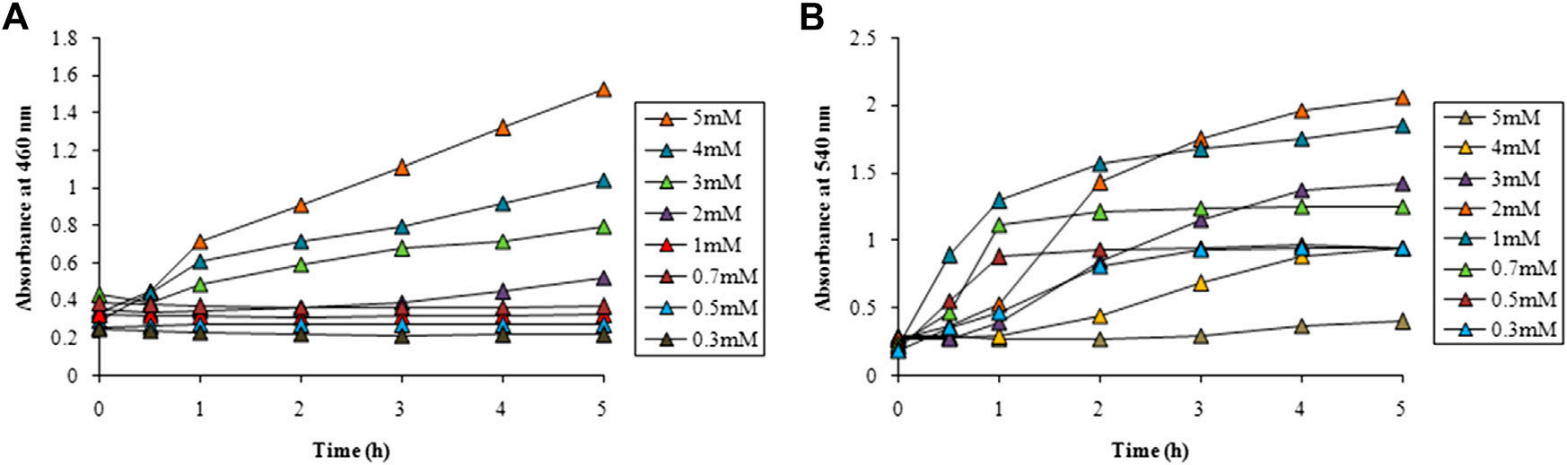
Time course of nanoparticle synthesis using LFLE at 40°C with **(A)** different concentrations of AgNO_3_ and **(B)** HAuCl_4_. The difference in synthesis is significant among different concentrations and time points with *p* < 0.05 by two factor ANOVA.

Optimization studies for AgNPs and AuNPs were carried out at 460 nm and 540 nm, respectively which were their absorbance maxima as revealed in the UV-visible spectroscopy of the particles concerned. Various parameters such as, metal precursor salt concentration, reaction temperature and LFLE concentration were optimized to get maximum rate of synthesis. Figure 2A shows the effect of AgNO_3_ concentration on the synthesis of AgNPs with time. Till 1 mM concentration no synthesis was observed up to 5 h while with 2 mM AgNO_3_ a slight increase was observed only after 3 h. Further increase in the AgNO_3_ concentration exhibited significant increase in the synthesis of AgNPs maximum being at 5 mM. Hence, for further optimization 5 mM of AgNO_3_ was selected. In case of AuNPs, 0.3 mM HAuCl_4_ showed negligible synthesis while further increase in the concentration of HAuCl_4_ resulted in steady rise in the rate of synthesis. With 1 mM of HAuCl_4_, synthesis was maximum till 3 h followed to which 2 mM showed an increased AuNPs production as evident from Figure 2B.

From the above results it is evident that the concentration of metal salts played a critical role in determining the speed of the nanoparticle synthesis. Concentration of AgNO_3_ above 1 mM facilitated better synthesis of AgNPs. Our results are well in agreement where extracts of *Carum carvi* could synthesize AgNPs with higher concentrations (10 mM) of AgNO_3_ (Nasiri and Nasiri, 2016). Similarly, Atunola et al. (2017) reported that 100 mM AgNO_3_ was required for synthesis of AgNPs by the extracts of *Allium sativum L.* (garlic), *Zingiber officinale* Rosc. (ginger), and *Capsicum frutescens L.* (cayenne pepper). This signifies that LFLE requires lower concentration of AgNO_3_ as compared to other phytogenic routes and hence is more economical as well. On the other hand 1 mM and 2 mM of HAuCl_4_ were found to be suitable for synthesis of AuNPs which is identical to the synthesis reported using extracts of *Litchi chinensis* and *Platanus orientalis* (Shende et al., 2017; Shende et al., 2018). It should be noted that reaction time can be remarkably shortened by increasing higher concentration of the metal ions if the quantity of the reducing phytochemicals in plant extract is not sufficiently high. Moreover, altering the metal ion concentration may modulate the morphologies and applications of the biogenic nanoparticles (Dhand et al., 2016; Zulfiqar et al., 2019).

**FIGURE 3:**
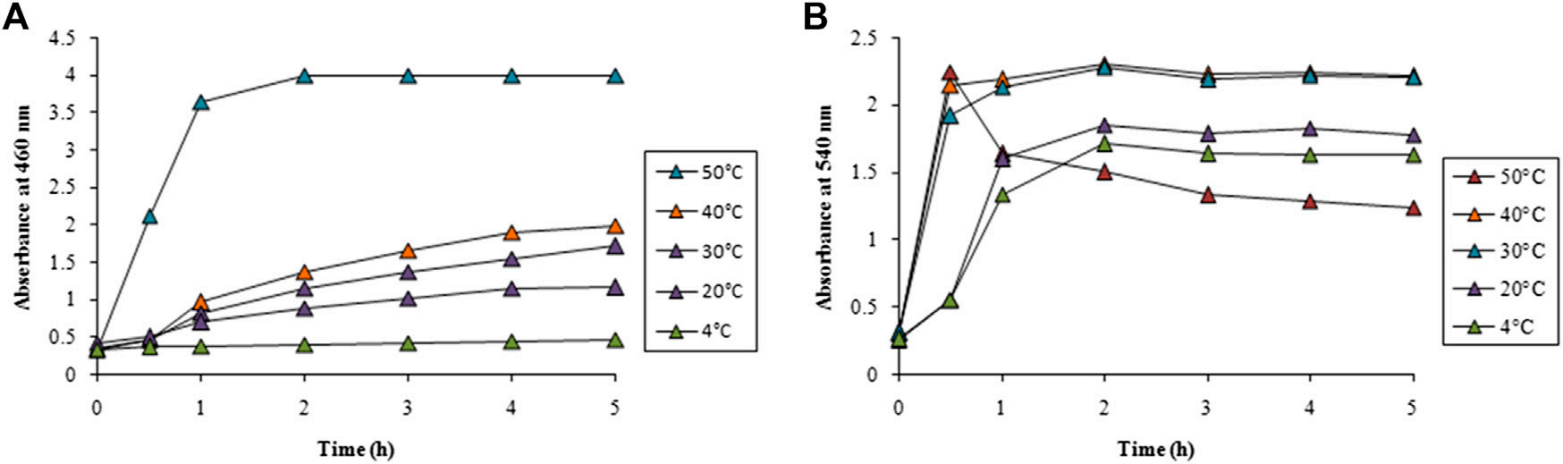
Time course of nanoparticle synthesis using LFLE at different reaction temperatures with **(A)** 5 mM AgNO_3_ and **(B)** 1 mM HAuCl_4_. The difference in synthesis is significant among different reaction temperatures and time points with *p* < 0.05 by two factor ANOVA.

Temperature had a pronounced effect on the rate of synthesis of both AgNPs and AuNPs as observed from Figure 3. No synthesis of AgNPs was observed at 4°C while gradual increase in the reaction temperature upto 40°C showed a steady rise in the synthesis. However, further increase of temperature to 50°C showed an abrupt enhancement in the rate of synthesis of AgNPs as observed in Figure 3A. It is interesting to note that the synthesis of AuNPs was almost instant even at 4°C that also increased with the increase in temperature. Beyond 30°C the rate of synthesis was identical and completed well before 1 h followed to which a plateau was observed as evident from Figure 3B.

**FIGURE 4:**
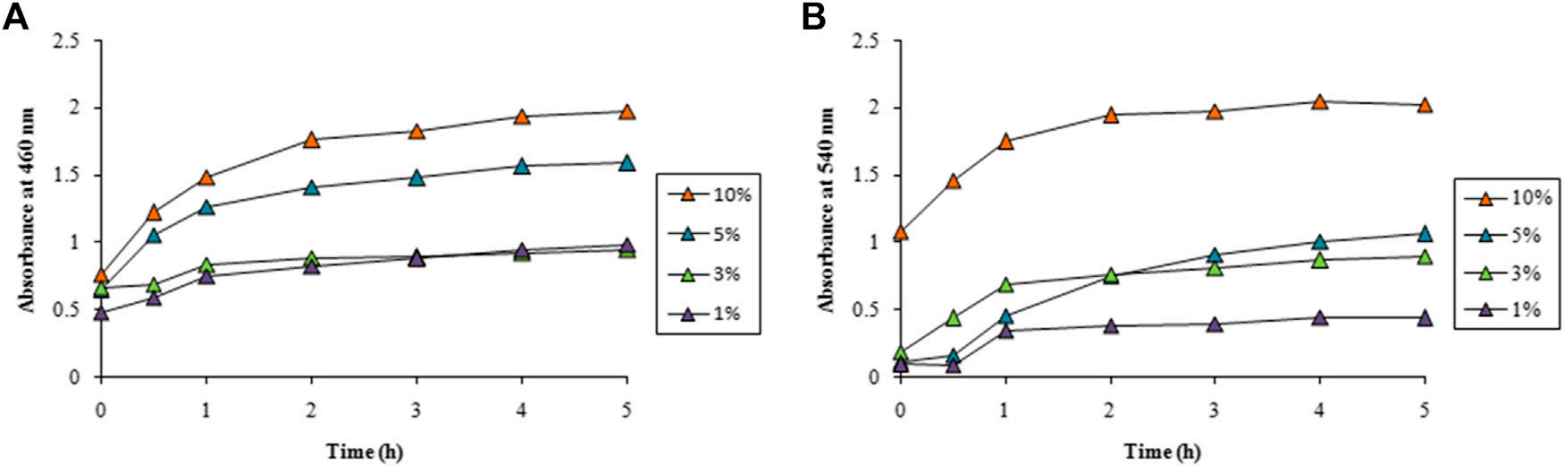
Time course of nanoparticle synthesis using different concentrations of LFLE at 40°C with **(A)** 5 mM AgNO_3_ and **(B)** 1 mM HAuCl_4_. The difference in synthesis is significant among different concentrations of LFLE and time points with *p* < 0.05 by two factor ANOVA.

LFLE concentration also exhibited a significant role in determining the speed of the synthesis. Very slow rate of AgNPs synthesis was achieved with 1 and 3% of LFLE which increased with increase in the LFLE concentration upto 10% as illustrated in Figure 4A. A distinct gradual increase in the rate of AuNPs on increasing the LFLE concentration from 1% to 10% was observed as depicted in Figure 4B.

Spectral data for optimization study is available in the Supplementary data (Supplementary Figures S1–S34). The concentration of LFLE also had pronounced effect on the synthesis of both AgNPs and AuNPs. As stated earlier, LFLE is a rich cocktail of diverse phytochemicals such as tannins, terpenoids, flavonoids, ketones, aldehydes, amides, and carboxylic acids that are assumed to donate electrons for reduction of Ag^1+^ to Ag^0^ (Prabhu and Poulose, 2012; Srikar et al., 2016). Similarly, water-soluble plant metabolites, such as proteins, and reducing sugars were mainly responsible for the biosynthesis of the metal nanoparticles by *Foeniculum vulgare* and *Dioscorea bulbifera* (Sulthana and Rajanikanth, 2018; Jamdade et al., 2019). Morphological features of the phytogenic nanoparticles were reported to be dependent on the concentrations of *Azadirachta indica* and *Cassia auriculata* L extracts (Roy et al., 2017; Bhuvaneswari et al., 2019). Our results are well in agreement where Chandraker et al. (2022a) showed that higher temperature (80°C) facilitated the synthesis of AgNPs using *Thalictrum foliolosum* DC leaf extract (TFLE). Optimization studies revealed that reaction time of 80 min, 2 ml TFLE, and 2 mM AgNO_3_ were ideal for maximum synthesis of AgNPs. Likewise the optimum temperature, pH, leaf extract concentration and AgNO_3_ concentration were 60°C, 8, 2 ml and 2 mM, respectively during synthesis of AgNPs employing *Rubia cordifolia L.* leaf extract. This signifies that the higher temperature can enhance the synthesis of AgNPs (Chandraker et al., 2022b). The yield of AgNPs and AuNPs were calculated as 40 mg and 30 mg from 100 ml reaction mixture, apparent cost of which were 35 (INR) and 311.5 (INR), respectively.

### High resolution transmission electron microscope, energy dispersive spectroscopy, dynamic light scattering analyses

**FIGURE 5:**
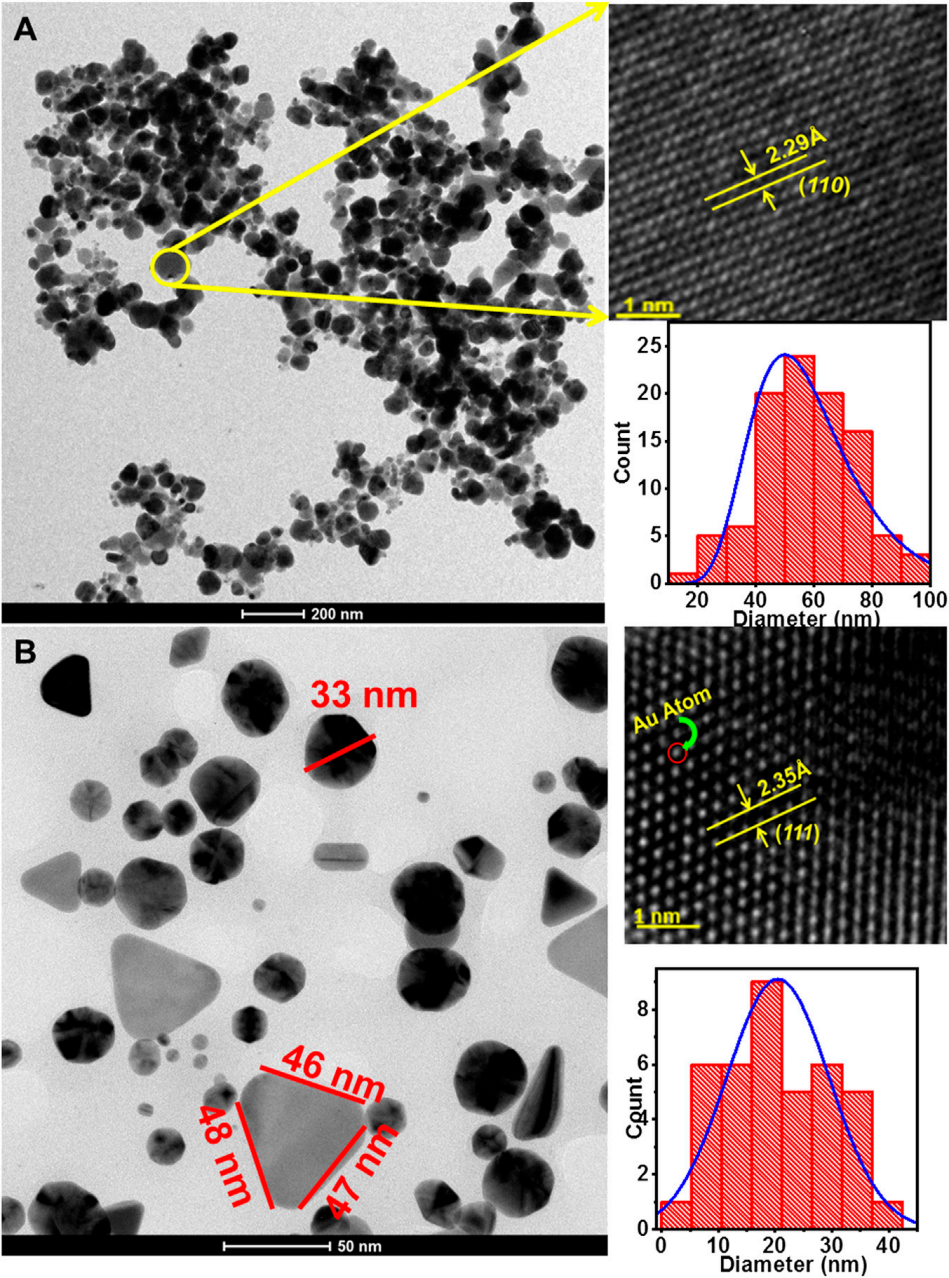
High-resolution transmission electron micrographs of nanoparticles synthesized by LFLE. **(A)** Spherical and irregular AgNPs synthesized by LFLE, **(B)** anisotropic AuNPs synthesized by LFLE showing triangular, spherical, rod shaped and blunt ended hexagonal shapes; their upper and lower insets showing the magnified HRTEM image and histogram plot of the particle distribution, respectively.

The size and shape of the bioreduced AgNPs and AuNPs were elucidated with the help of HRTEM. The HRTEM images of the bio-synthesized AgNPs and AuNPs are shown in Figures 5A,B, respectively. The bioreduced AgNPs as illustrated in Figure 5A were mostly spherical and irregular in shape. The particles were attached to each other in discrete clusters. The average diameter of the AgNPs was calculated from the histogram plot as shown in the lower right inset of Figure 5A and was found to be ∼50 ± 15 nm. Figure 5A shows smaller AgNPs attached to the surface of the larger particles indicating the increase in size due to fusion of initially formed smaller AgNPs. Distinct lattice fringes were observed on the surface of the AgNPs in the magnified HRTEM image of the selected portion of Figure 5A as shown in the upper right inset. The inter-planer *d*-spacing of 2.29 Å corresponds to (*110*) plane of AgNPs. Anisotropic AuNPs with varied shapes and sizes were formed by LFLE as evident from Figure 5B. The phytogenic AuNPs showed spherical, triangular, blunt ended hexagonal and even rod shaped particles. The average diameter of the spherical AuNPs was ∼22 ± 20 nm as shown in the histogram plot in the lower right inset of Figure 5B.

The length of the sides of the triangular AuNPs was ∼46 nm. The AuNPs were separate and stable with no sign of agglomeration. Distinct lattice fringes on the surface of the spherical AuNPs was observed in the magnified HRTEM image of the selected portion of Figure 5B as shown in the upper right inset and it is observed that the inter-planer *d*-spacing was 2.35 Å which corresponds to (*111*) plane of AuNPs.

**FIGURE 6:**
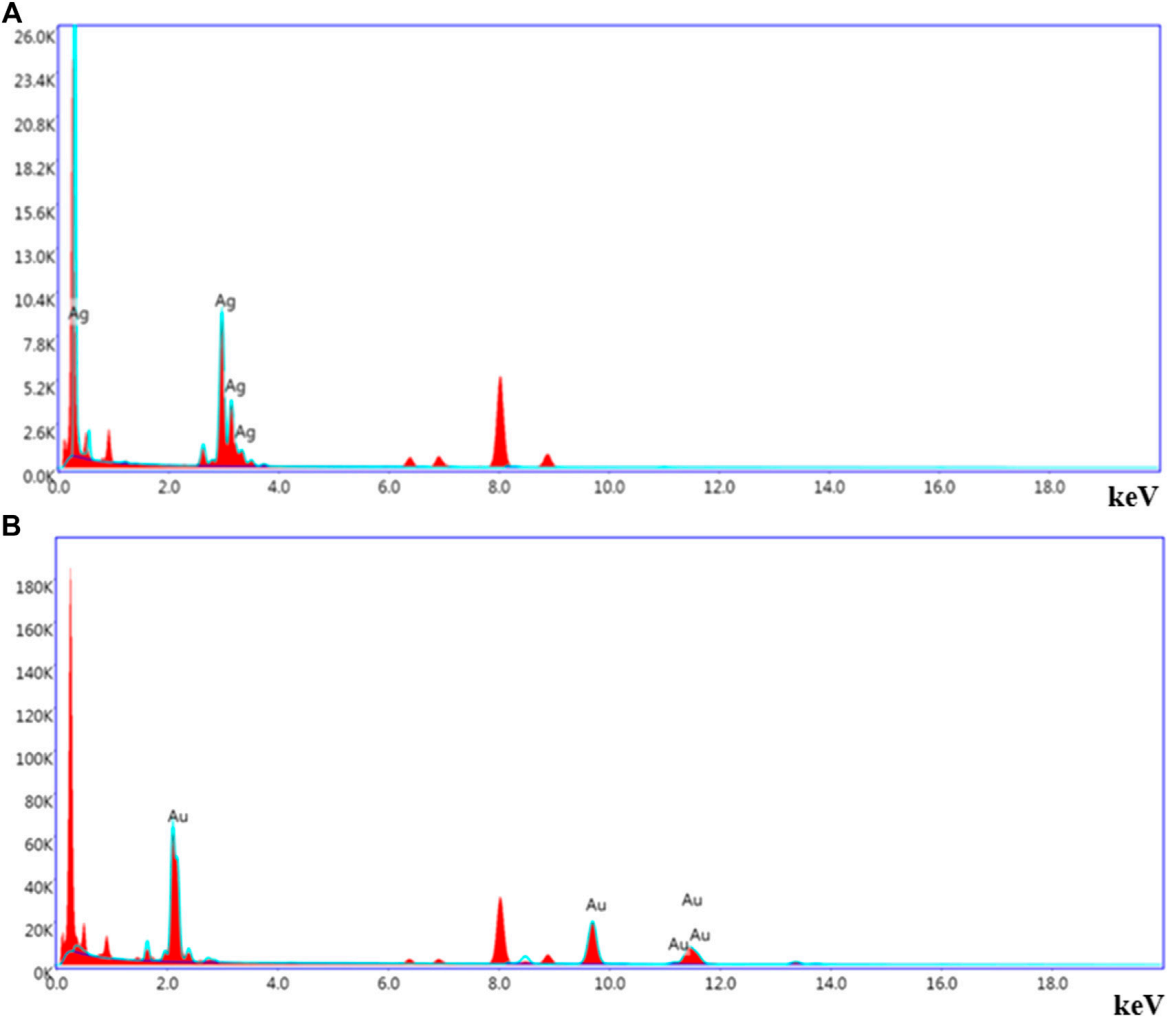
Representative spot EDS profile. **(A)** AgNPs and **(B)** AuNPs.

**FIGURE 7:**
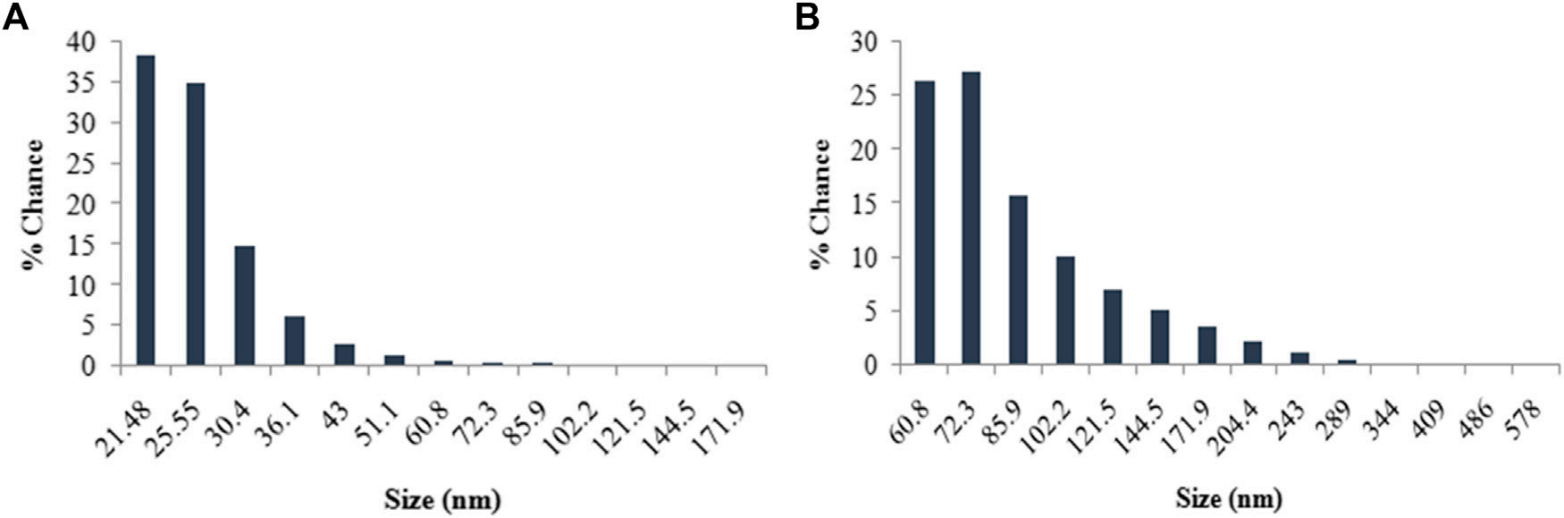
Histogram of size distribution of nanoparticles synthesized by LFLE. **(A)** AgNPs and **(B)** AuNPs.

The energy dispersive spectra in Figure 6 confirmed the presence of elemental silver and gold in the LFLE synthesized AgNPs and AuNPs, respectively. The hydrodynamic size distribution of the colloidal AgNPs and AuNPs as revealed by DLS in Figure 7 is in close agreement with the HRTEM results. The average particle size of synthesized AgNPs and AuNPs were 34.8 nm and 140.8 nm, respectively that were larger compared to HRTEM results. This might be attributed to the agglomeration of the smaller particles into larger clusters with time. The zeta potential of the biogenic AgNPs and AuNPs were 0.67 mV and 5.70 mV, respectively which indicate the higher stability of AuNPs.

The particle size achieved in the LFLE mediated synthesis of AgNPs was similar to that achieved with leaf and root extract of *Ricinus communis* that also gave spherical particles in range between 29 and 38 nm of diameter (Gul et al., 2021). Another study with onion peel as a reducing agent synthesized AgNPs at 90°C which were spherical in shape with 12.5 nm particle size (Abdullah et al., 2021). Earlier studies on phytofabrication of AgNPs using *Bryophyllum pinnatum* leaf extract also showed predominance of similar spherical shaped particles with an average size of ∼15 nm (Chandraker et al., 2021a). However, anisotropic polydispersed AuNPs were synthesized by LFLE which is in close agreement with triangular, pentagonal, and hexagonal shaped AuNPs synthesized using extracts from various plants such as blackberry, blueberry, pomegranate, and turmeric (Nadagouda et al., 2014; Castillo-Henríquez et al., 2020). Zeta potential reflects the effective charge on the surface of nanoparticles. The stable and discrete AuNPs unlike the agglomerated AgNPs was attributed to their higher zeta value (Leroy et al., 2011). The fact was also reflected in the hydrodynamic size as confirmed using DLS.

### X-ray diffraction pattern analyses

**FIGURE 8:**
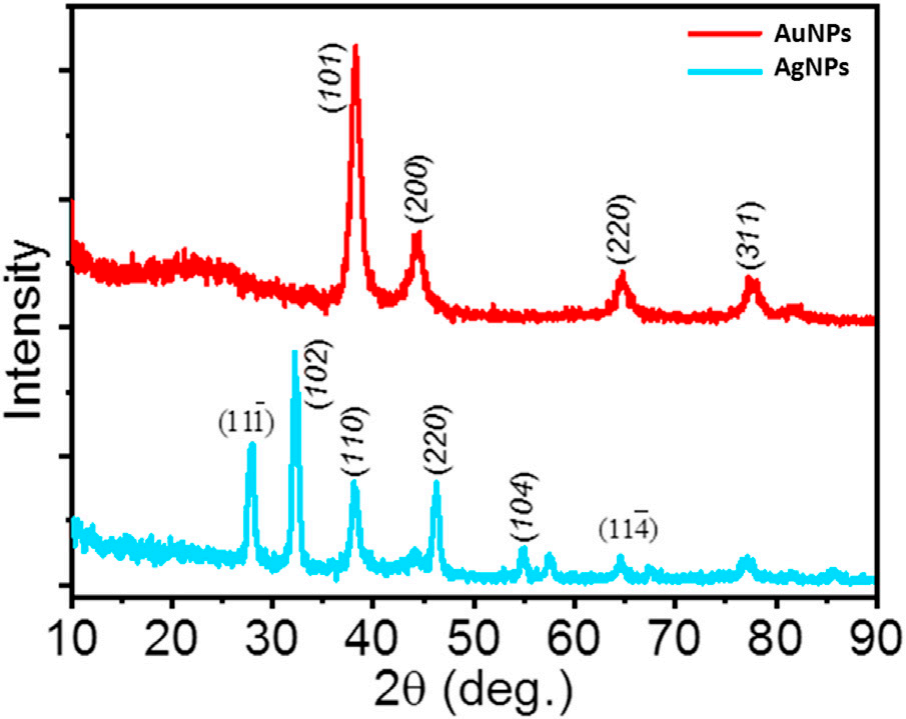
XRD spectra of AgNPs and AuNPs.

The XRD patterns of the AgNPs and AuNPs are shown in Figure 8. The XRD spectrum of AgNPs reveal a large number of peaks at 2θ value of 27.90^°^, 32.30^°^, 38.10^°^, 46.30^°^, 54.90^°^, and 64.40^°^ corresponding to $\left(11\bar{1}\right)$ , (*102*), (*110*), (*220*), (*104*), and $\left(11\bar{4}\right)$ planes of silver, respectively. Likewise, the XRD spectrum of AuNPs reveal a large number of peaks at 2θ value of 38.10^°^, 44.30^°^, 64.70^°^, and 77.40^°^ corresponding to (*101*), (*200*), (*220*), and (*311*) planes of gold, respectively.

### Fourier transform infrared spectroscopy spectral analyses

**FIGURE 9:**
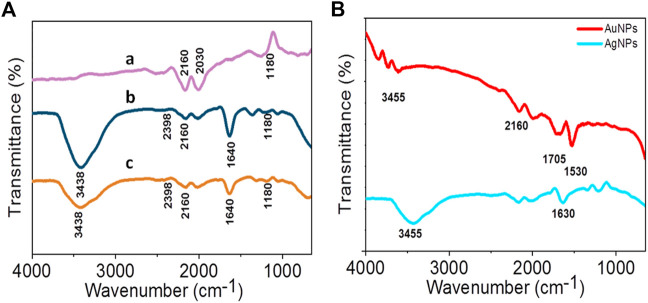
**(A)** FTIR spectra of LFLE after reduction of Au^3+^ **(a)** and Ag^1+^ **(b)** compared with before reduction **(c)**; **(B)** FTIR spectra of AgNPs and AuNPs.

The FTIR transmittance spectra of LFLE before and after synthesis of AuNPs and AgNPs showed different vibrations and bond stretching as observed in Figure 9. The absorbance bands in LFLE at 3438 cm^−1^, 2398 cm^−1^, 2160 cm^−1^, 1640 cm^−1^, and 1180 cm^−1^ are associated with the stretch vibrations of N-H stretching, O=C=O stretching, C-H bending, C=O stretching and C-O stretching, respectively (Krueger and Smith, 1967; Goudarzi et al., 2016; Md Salim et al., 2021). The LFLE showed aliphatic primary amine which is supported by the presence of a strong peak approximately at 3438 cm^−1^. This sharp peak representing N–H bond was not seen in LFLE after bioreduction of HAuCl_4_. This indicates that primary amines are mainly responsible for the reduction of Au^3+^ into AuNPs. We could observe minor C-O stretching and the presence of secondary alcohol in the FTIR of AuNPs at 1180 cm^−1^. AgNPs showed sharp peak stretching of N-H bond on 3438 cm^−1^. The presence of peaks at 2398 cm^−1^, 2160 cm^−1^, 1640 cm^−1^ and 1180 cm^−1^ indicated that the AgNPs may be surrounded by amines and hydroxyl groups as a stabilizing agent because the peaks indicate symmetric minor stretching (Goudarzi et al., 2016).

It is interesting to note that various functional groups of the phytoconstituents in LFLE were responsible in the reduction of the metal ions to AgNPs and AuNPs as revealed by FTIR. Further, some functional groups were also associated on the surface of the phytogenic nanoparticles that might play a key role in the capping. The slight band shift might be attributed to transition of the phytochemicals such as alkaloids, flavonoids, tannins, terpenes and quinones from free to nanoparticle bound form. These shifts rationalize their involvement in the metal reduction and formation of the nanoparticles (Ynalvez and Compean, 2014). The anisotropy was attributed to the obvious presence of diverse reducing and capping agents. Hence, in order to address the polydispersity issue pure phytochemicals like quercetin can be employed for synthesis of nanoparticles that can give better control over the size and shape of the phytogenic nanoparticles (Jain and Mehata, 2017). Ghosh et al. (2020) reported the involvement of flavonoids, tannins, glycosides, and alkaloids as potential reducing, stabilizing and capping agents responsible in synthesis of stable copper nanoparticles (CuNPs) with average size of 10 ± 1 nm using a leaf extract from *Jatropha curcas*.

### Photocatalytic dye degradation

**FIGURE 10:**
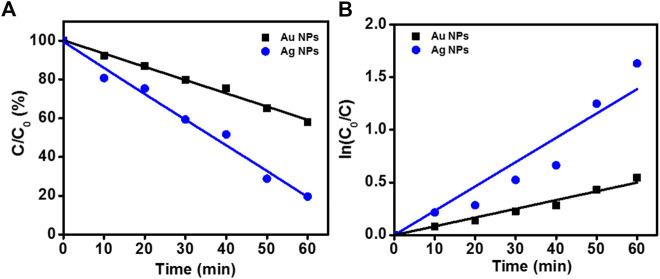
Degradation of RhB in presence of nanoparticles. **(A)** Photocatalytic RhB degradation by AgNPs and AuNPs under the UV light; **(B)** First-order kinetic constant for the degradation of RhB under UV-light.

The photocatalytic activity of samples is shown in Figure 10. The degradation of RhB under UV irradiation in presence of phytogenic AgNPs and AuNPs was monitored using an UV-visible spectrophotometer. The absorption maxima of RhB was centered at 553 nm. The main absorption peak steadily decreased and eventually approached the base line in both the cases as clearly evident from Figure 10A. The plot of ln (C_0_/C) vs. time for the catalytic degradation of RhB is shown in Figure 10B. The rate constants were calculated as 0.0231 s^−1^ and 0.00831 s^−1^, for AgNPs and AuNPs, respectively. The experimental data fits well with the first order kinetic model with 23.1 % and 31.7% degradation of RhB dye by AgNPs and AuNPs, respectively.

The degradation mechanism for metal nanoparticles has been widely studied. A possible mechanism for the degradation of organic dye in this study is given below where M represents the metallic element (Baruah et al., 2018; Khan et al., 2020)

$$M^{0}+hv\left(UV\right)\to MNPs\left(e_{CB-}+h_{VB+}\right)$$

M(hVB+)+H2O→M0+H+•O•H

$$M\left(e_{CB-}\right)+O_{2}\to MNPs+O_{2}^{•-}$$

$$O_{2}^{•-}+H^{+}\to HO_{2}^{•}$$

Dye+O•H→Degradation−products

$$Dye+h_{VB+}\to Degradation-products$$

$$Dye+e_{CB+}\to Degradation-products$$

The results of the present study exhibited a good catalytic potential of the synthesized AgNPs and AuNPs towards the reductive degradation of RhB dye which is in consensus with the previously addressed studies on phytogenic nanoparticles from extracts of *Trigonella foenum-graecum* seed, *Alpinia nigra* leaves, and *Cocos nucifera* (Baruah et al., 2018; Awad et al., 2021; Rajendran et al., 2021). It was speculated that the layer of the reducing agent on the surface of the biogenic nanoparticles may promote the effective adsorption of the dye molecules on to the nanoparticle surface. This in turn facilitates the oxidation-reduction between the active RhB dye and reducing agent that occurs more conveniently at a faster rate if the particles are smaller in size (Yazdi et al., 2020). Our results indicated that the reactivity of large surface area of AgNPs and AuNPs attributed to an efficient photocatalytic degradation of the RhB dye substantiating their promising role in elimination of dye pollution. Earlier reports have established that such oxidation-reduction reaction can occur due to involvement of intermediates such as hydroxyl radicals (David and Moldovan, 2020). Further, it was proposed that the irradiation mediated photocatalysis in dye might be due to the transfer of excited electrons from the valance band to the conduction band by the generation of electron hole pair. As an active oxidizing agent, the generated hydroxyl radical then degrades the dye to nontoxic products (carbon dioxide, water, etc.). Both AgNPs and AuNPs with high stability, conductivity and optical properties enable efficient trapping of photo excited electrons on the surface of photocatalytic material preventing the recombination of the electron hole pair (Rostami-Vartooni et al., 2016). Photocatalytic potential of nanoparticles under visible light was proposed to be attributed to their SPR associated collective oscillations of electrons promoting the interaction between the generated free radicals and molecular oxygen. Additionally, dye degradation can also involve the positive holes generated due to electron excitation (Marimuthu et al., 2020).

Biogenic nanoparticles are reported to show high catalytic activity due to their smaller size and stability (Ghosh et al., 2022a; Nitnavare et al., 2022). Photocatalytic activity of phytogenic nanoparticles was also reported by Chandraker et al. (2020) where anisotropic CuNPs synthesized by *Ageratum houstonianum* leaf extract had exhibited cubic, hexagonal, and rectangular shape, with average size of 80 nm. The phytogenic CuNPs effectively degraded Congo red dye within 2 h with a pseudo-first-order kinetics. The phytogenic nanoparticles with catalytic activity can be further coupled with solvent impregnated resin (SIR) for their potential application like removal and sensing of refractory pollutants such as dye, metal ions and phenolic compounds from industrial effluents (Tamang et al., 2022). Additionally, these nanoparticles can be functionalized with bioactive principles, drugs, targeting ligands and/or contrast agents for extending their applications as antimicrobial, anticancer, antioxidant, tissue engineering and theranostic agents (Chandraker et al., 2021b; Ghosh et al., 2022b).

## Conclusion

*L. frutescens* leaf extract mediated synthesis of AgNPs and AuNPs is an efficient, rapid and environmentally benign approach. This green route involves the naturally occurring bioactive molecules in the plant extract for reducing the metal ions to corresponding nanoparticles and their further stabilization. The spherical to irregular shaped AgNPs with an average diameter of ∼50 ± 15 nm were synthesized within 5 h having elemental Ag as confirmed by EDS. Polydispersed AuNPs were comprised of spherical, triangular, rod, and hexagonal shaped nanoparticles with an average size of ∼22 ± 20 nm. Optimization of various reaction parameters such as reaction time, metal salt concentration, temperature, and LFLE concentration showed their promising role in determining the rate of the synthesis. FTIR spectra identified the potential functional groups in LFLE that were involved in rapid reduction of Ag^+^ and Au^3+^ ions to Ag^0^ and Au^0^. The phytofabricated AgNPs and AuNPs exhibited catalytic degradation of RhB dye rationalizing their promising applications in treatment of dye contaminated industrial effluent. Based on the remarkable photocatalytic activities of AgNPs and AuNPs it is hereby recommended for its uses in biomedical applications and tissue engineering with elaborated research with respect to photothermal and photodynamic therapy for controlling biofilm associated infections and cancer.

## Data Availability

The original contributions presented in the study are included in the article/Supplementary Material, further inquiries can be directed to the corresponding authors.
