# Supplementary material for: Leucophyllum frutescens mediated synthesis of silver and gold nanoparticles for catalytic dye degradation
Source: Front Chem. 2022 Sep 29;10:932416. doi: 10.3389/fchem.2022.932416 (PMC9557002; doi:10.3389/fchem.2022.932416)
Supplement: Supplementary file 1 [file DataSheet1.doc]

## *Leucophyllum frutescens* mediated synthesis of silver and gold nanoparticles for catalytic dye degradation

### Bansuri Gami,^1^ Khalida Bloch,^1^ Shahansha M. Mohammed,^2^ Srikanta Karmakar,^3^ Satyajit Shukla,^2,4^ Adersh Asok,^4,5^ Sirikanjana Thongmee,^6^* and Sougata Ghosh^1,6^*

## ^1^Department of Microbiology, School of Science, RK University, Rajkot-360020, Gujarat, India;

^2^Functional Materials Section (FMS), Materials Science and Technology Division (MSTD), CSIR-National Institute for Interdisciplinary Science and Technology (NIIST), Council of Scientific and Industrial Research (CSIR), Industrial Estate P. O., Pappanamcode Thiruvananthapuram – 695019, Kerala, India;

## ^3^Department of Polymer Science and Technology, Calcutta University, Kolkata-700009, West Bengal, India;

^4^Academy of Scientific and Innovative Research (AcSIR), Ghaziabad - 201002, India;

^5^Photosciences and Photonics Section, Chemical Sciences and Technology Division (CSTD), CSIR-National Institute for Interdisciplinary Science and Technology (NIIST)

Council of Scientific and Industrial Research (CSIR) Industrial Estate P. O., Pappanamcode Thiruvananthapuram – 695019, Kerala, India; and

## ^6^Department of Physics, Faculty of Science, Kasetsart University, Bangkok-10900, Thailand

Supplementary figures

| Figure number | Title |
| --- | --- |
| **Figure S1** | UV-visible spectra recorded as a function of reaction time for AgNPs synthesis using LFLE with 0.3 mM AgNO_3_. |
| **Figure S2** | UV-visible spectra recorded as a function of reaction time for AgNPs synthesis using LFLE with 0.5 mM AgNO_3_. |
| **Figure S3** | UV-visible spectra recorded as a function of reaction time for AgNPs synthesis using LFLE with 0.7 mM AgNO_3_. |
| **Figure S4** | UV-visible spectra recorded as a function of reaction time for AgNPs synthesis using LFLE with 1 mM AgNO_3_. |
| **Figure S5** | UV-visible spectra recorded as a function of reaction time for AgNPs synthesis using LFLE with 2 mM AgNO_3_. |
| **Figure S6** | UV-visible spectra recorded as a function of reaction time for AgNPs synthesis using LFLE with 3 mM AgNO_3_. |
| **Figure S7** | UV-visible spectra recorded as a function of reaction time for AgNPs synthesis using LFLE with 4 mM AgNO_3_. |
| **Figure S8** | UV-visible spectra recorded as a function of reaction time for AgNPs synthesis using LFLE with 5 mM AgNO_3_. |
| **Figure S9** | UV-visible spectra recorded as a function of reaction time for AuNPs synthesis using LFLE with 0.3 mM HAuCl_4_. |
| **Figure S10** | UV-visible spectra recorded as a function of reaction time for AuNPs synthesis using LFLE with 0.5 mM HAuCl_4_. |
| **Figure S11** | UV-visible spectra recorded as a function of reaction time for AuNPs synthesis using LFLE with 0.7 mM HAuCl_4_. |
| **Figure S12** | UV-visible spectra recorded as a function of reaction time for AuNPs synthesis using LFLE with 1 mM HAuCl_4_. |
| **Figure S13** | UV-visible spectra recorded as a function of reaction time for AuNPs synthesis using LFLE with 2 mM HAuCl_4_. |
| **Figure S14** | UV-visible spectra recorded as a function of reaction time for AuNPs synthesis using LFLE with 3 mM HAuCl_4_. |
| **Figure S15** | UV-visible spectra recorded as a function of reaction time for AuNPs synthesis using LFLE with 4 mM HAuCl_4_. |
| **Figure S16** | UV-visible spectra recorded as a function of reaction time for AuNPs synthesis using LFLE with 5 mM HAuCl_4_. |
| **Figure S17** | UV-visible spectra recorded as a function of reaction time for AgNPs synthesis using LFLE with 5 mM AgNO_3_ at 4 °C. |
| **Figure S18** | UV-visible spectra recorded as a function of reaction time for AgNPs synthesis using LFLE with 5 mM AgNO_3_ at 20 °C. |
| **Figure S19** | UV-visible spectra recorded as a function of reaction time for AgNPs synthesis using LFLE with 5 mM AgNO_3_ at 30 °C. |
| **Figure S20** | UV-visible spectra recorded as a function of reaction time for AgNPs synthesis using LFLE with 5 mM AgNO_3_ at 40 °C. |
| **Figure S21** | UV-visible spectra recorded as a function of reaction time for AgNPs synthesis using LFLE with 5 mM AgNO_3_ at 50 °C. |
| **Figure S22** | UV-visible spectra recorded as a function of reaction time for AuNPs synthesis using LFLE with 1 mM HAuCl_4_ at 4 °C. |
| **Figure S23** | UV-visible spectra recorded as a function of reaction time for AuNPs synthesis using LFLE with 1 mM HAuCl_4_ at 20 °C. |
| **Figure S24** | UV-visible spectra recorded as a function of reaction time for AuNPs synthesis using LFLE with 1 mM HAuCl_4_ at 30 °C. |
| **Figure S25** | UV-visible spectra recorded as a function of reaction time for AuNPs synthesis using LFLE with 1 mM HAuCl_4_ at 40 °C. |
| **Figure S26** | UV-visible spectra recorded as a function of reaction time for AuNPs synthesis using LFLE with 1 mM HAuCl_4_ at 50 °C. |
| **Figure S27** | UV-visible spectra recorded as a function of reaction time for AgNPs synthesis with 5 mM AgNO_3_ using 1% LFLE. |
| **Figure S28** | UV-visible spectra recorded as a function of reaction time for AgNPs synthesis with 5 mM AgNO_3_ using 3% LFLE. |
| **Figure S29** | UV-visible spectra recorded as a function of reaction time for AgNPs synthesis with 5 mM AgNO_3_ using 5% LFLE. |
| **Figure S30** | UV-visible spectra recorded as a function of reaction time for AgNPs synthesis with 5 mM AgNO_3_ using 10% LFLE. |
| **Figure S31** | UV-visible spectra recorded as a function of reaction time for AuNPs synthesis with 1 mM HAuCl_4_ using 1% LFLE. |
| **Figure S32** | UV-visible spectra recorded as a function of reaction time for AuNPs synthesis with 1 mM HAuCl_4_ using 3% LFLE. |
| **Figure S33** | UV-visible spectra recorded as a function of reaction time for AuNPs synthesis with 1 mM HAuCl_4_ using 5% LFLE. |
| **Figure S34** | UV-visible spectra recorded as a function of reaction time for AuNPs synthesis with 1 mM HAuCl_4_ using 10% LFLE. |


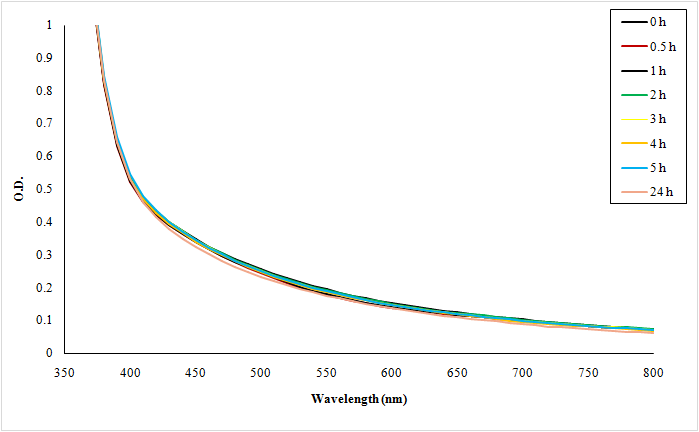


**Figure S1.** UV-visible spectra recorded as a function of reaction time for AgNPs synthesis using LFLE with 0.3 mM AgNO_3_.


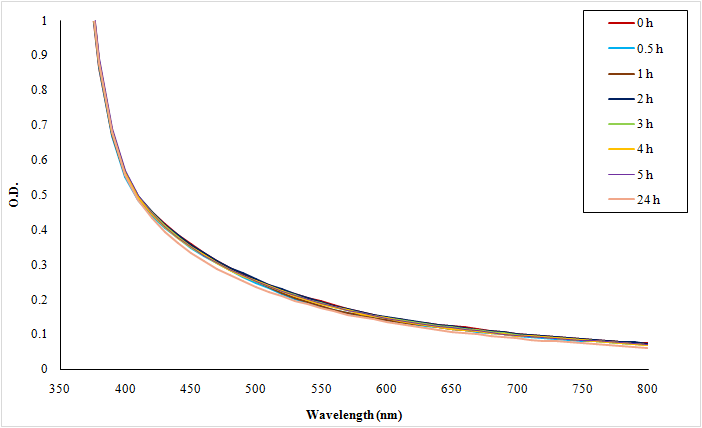


**Figure S2.** UV-visible spectra recorded as a function of reaction time for AgNPs synthesis using LFLE with 0.5 mM AgNO_3_.


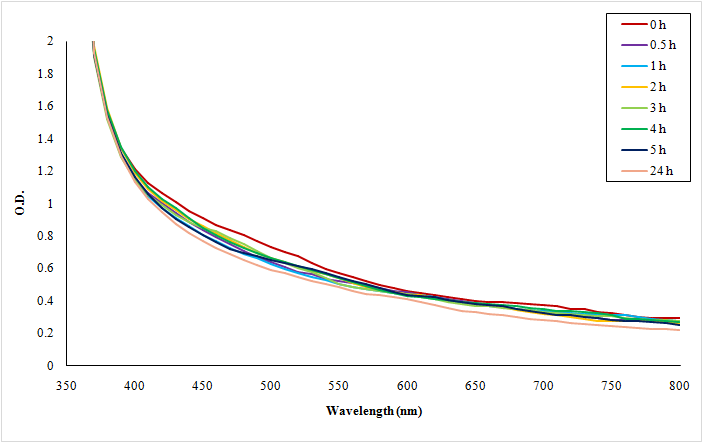


**Figure S3.** UV-visible spectra recorded as a function of reaction time for AgNPs synthesis using LFLE with 0.7 mM AgNO_3_.


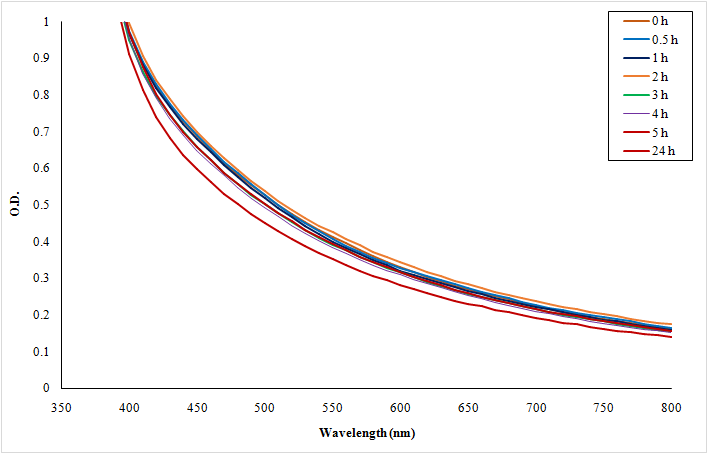


**Figure S4.** UV-visible spectra recorded as a function of reaction time for AgNPs synthesis using LFLE with 1 mM AgNO_3_.


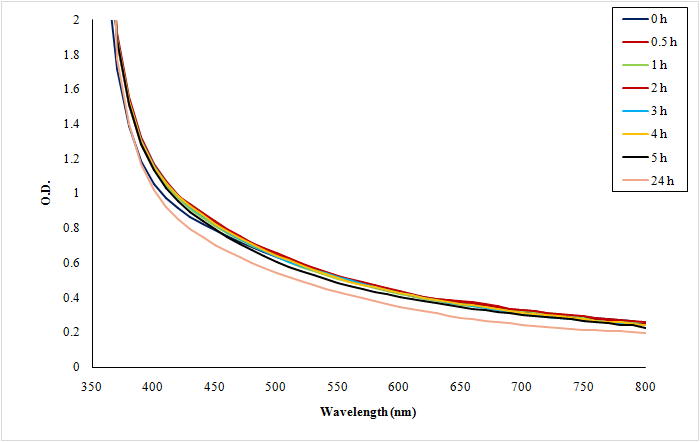


**Figure S5.** UV-visible spectra recorded as a function of reaction time for AgNPs synthesis using LFLE with 2 mM AgNO_3_.


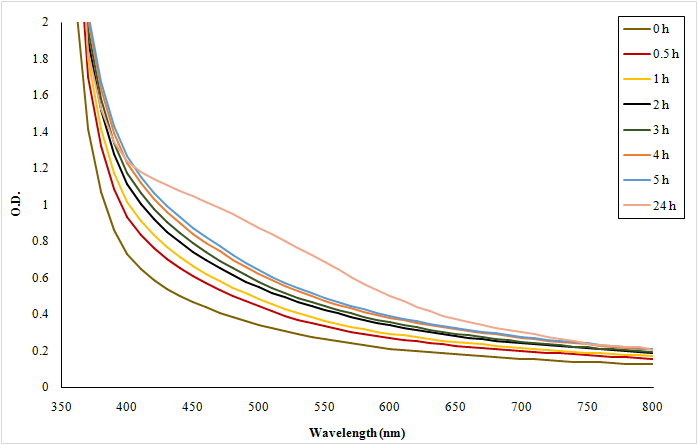


**Figure S6.** UV-visible spectra recorded as a function of reaction time for AgNPs synthesis using LFLE with 3 mM AgNO_3_.


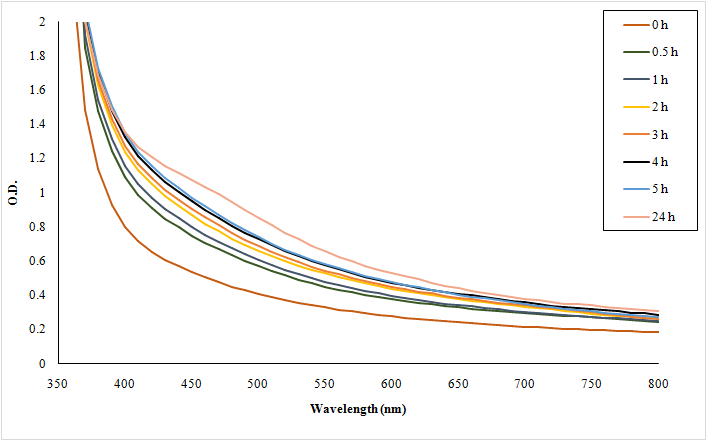


**Figure S7.** UV-visible spectra recorded as a function of reaction time for AgNPs synthesis using LFLE with 4 mM AgNO_3_.


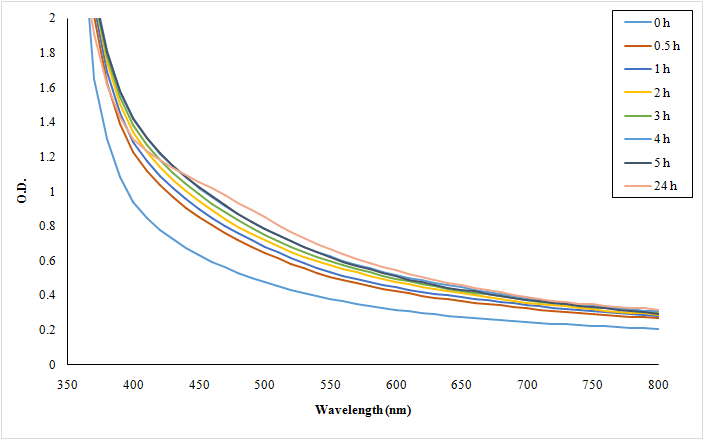


**Figure S8.** UV-visible spectra recorded as a function of reaction time for AgNPs synthesis using LFLE with 5 mM AgNO_3_.


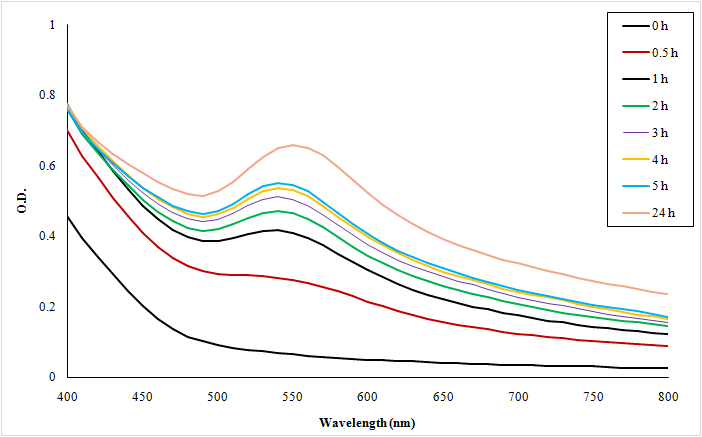


**Figure S9.** UV-visible spectra recorded as a function of reaction time for AuNPs synthesis using LFLE with 0.3 mM HAuCl_4_.


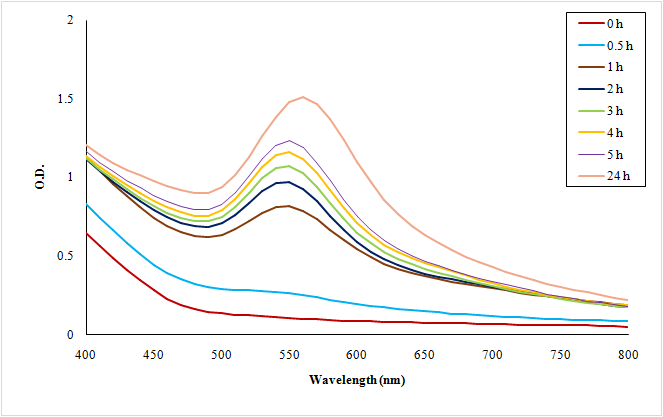


**Figure S10.** UV-visible spectra recorded as a function of reaction time for AuNPs synthesis using LFLE with 0.5 mM HAuCl_4_.


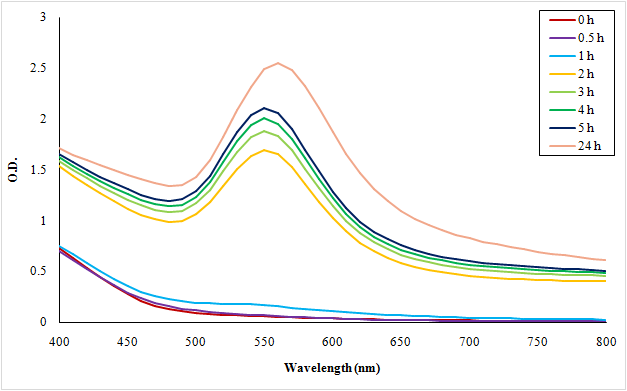


**Figure S11.** UV-visible spectra recorded as a function of reaction time for AuNPs synthesis using LFLE with 0.7 mM HAuCl_4_.


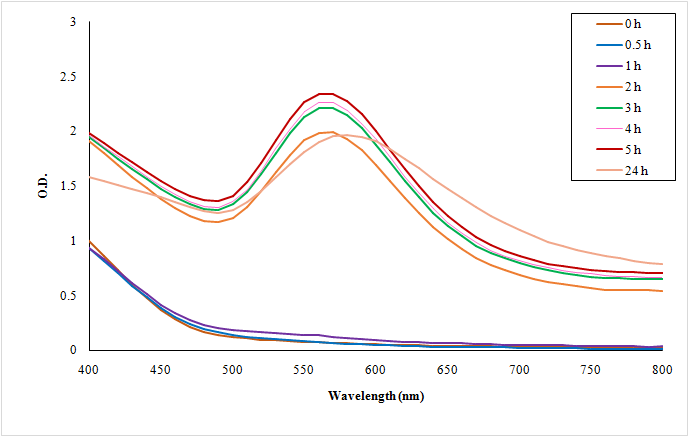


**Figure S12.** UV-visible spectra recorded as a function of reaction time for AuNPs synthesis using LFLE with 1 mM HAuCl_4_.


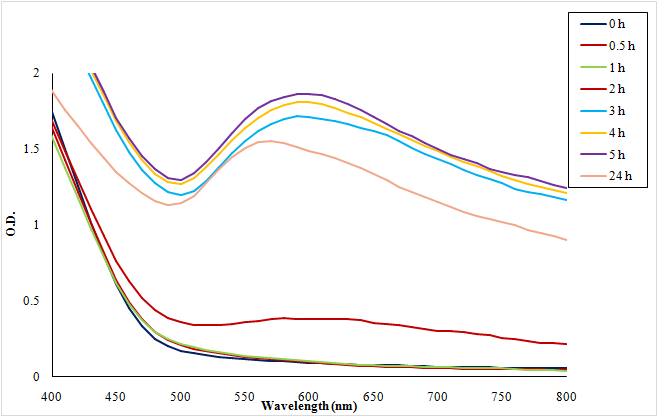


**Figure S13.** UV-visible spectra recorded as a function of reaction time for AuNPs synthesis using LFLE with 2 mM HAuCl_4_.


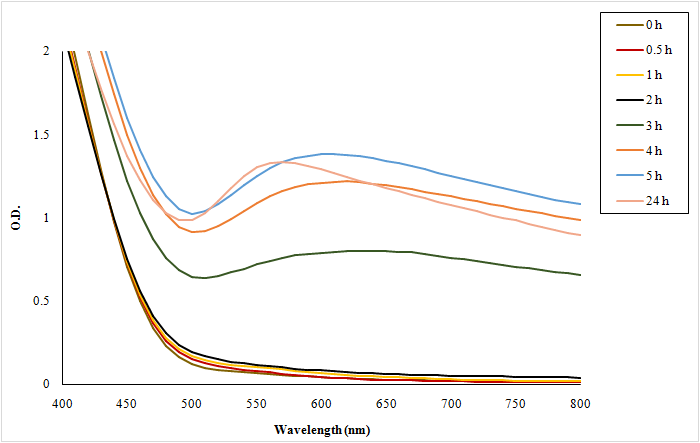


**Figure S14.** UV-visible spectra recorded as a function of reaction time for AuNPs synthesis using LFLE with 3 mM HAuCl_4_.


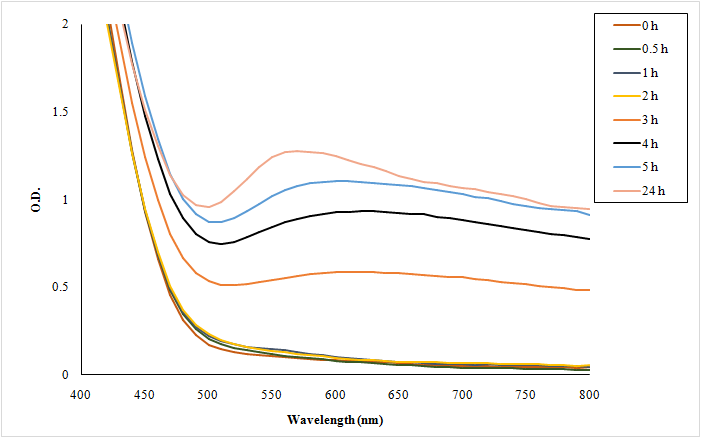


**Figure S15.** UV-visible spectra recorded as a function of reaction time for AuNPs synthesis using LFLE with 4 mM HAuCl_4_.


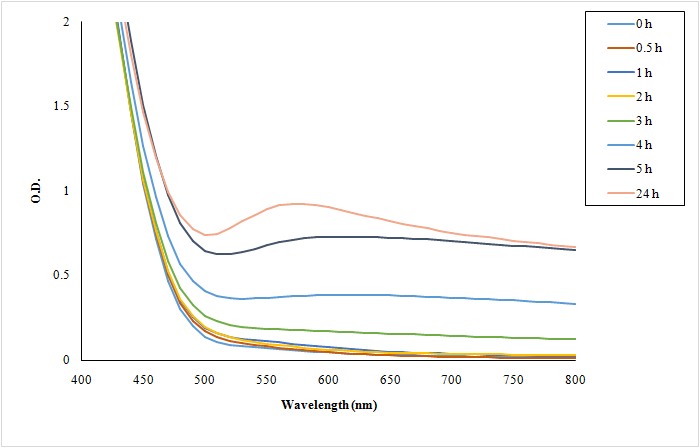


**Figure S16.** UV-visible spectra recorded as a function of reaction time for AuNPs synthesis using LFLE with 5 mM HAuCl_4_.


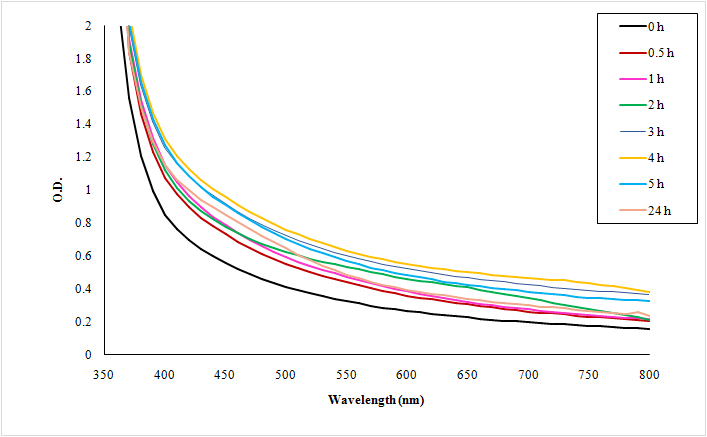


**Figure S17.** UV-visible spectra recorded as a function of reaction time for AgNPs synthesis using LFLE with 5 mM AgNO_3_ at 4 °C.


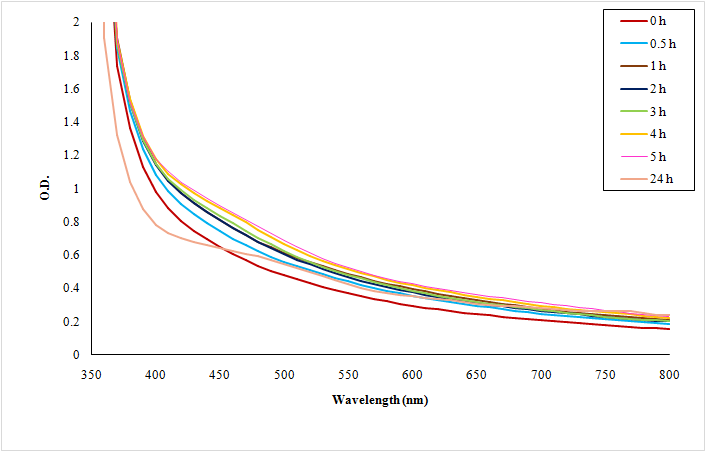


**Figure S18.** UV-visible spectra recorded as a function of reaction time for AgNPs synthesis using LFLE with 5 mM AgNO_3_ at 20 °C.


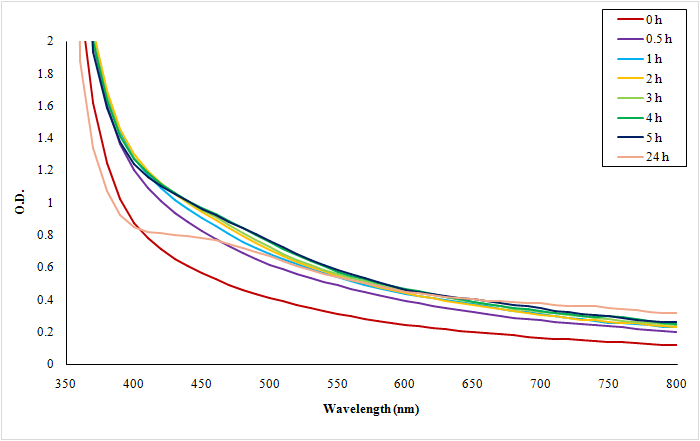


**Figure S19.** UV-visible spectra recorded as a function of reaction time for AgNPs synthesis using LFLE with 5 mM AgNO_3_ at 30 °C.


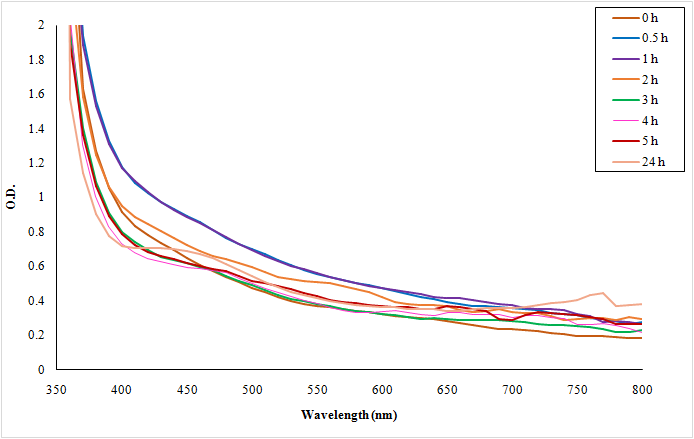


**Figure S20.** UV-visible spectra recorded as a function of reaction time for AgNPs synthesis using LFLE with 5 mM AgNO_3_ at 40 °C.


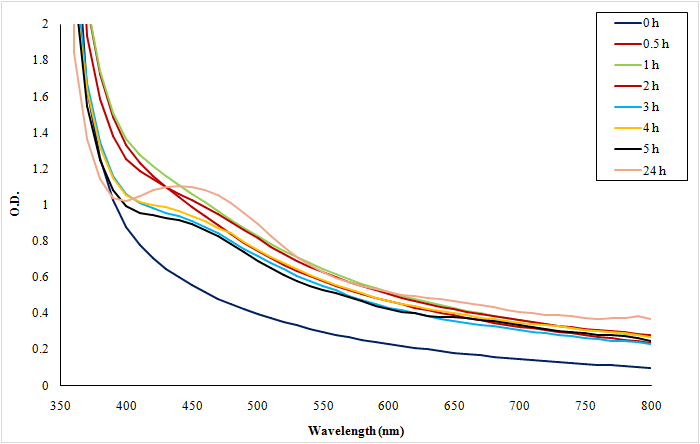


**Figure S21.** UV-visible spectra recorded as a function of reaction time for AgNPs synthesis using LFLE with 5 mM AgNO_3_ at 50 °C.


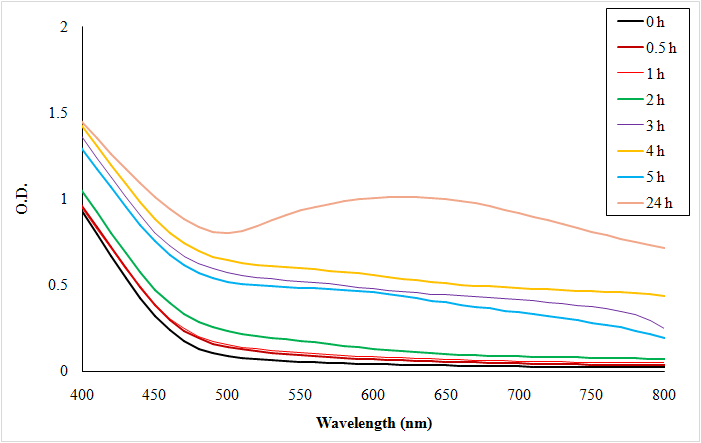


**Figure S22.** UV-visible spectra recorded as a function of reaction time for AuNPs synthesis using LFLE with 1 mM HAuCl_4_ at 4 °C.


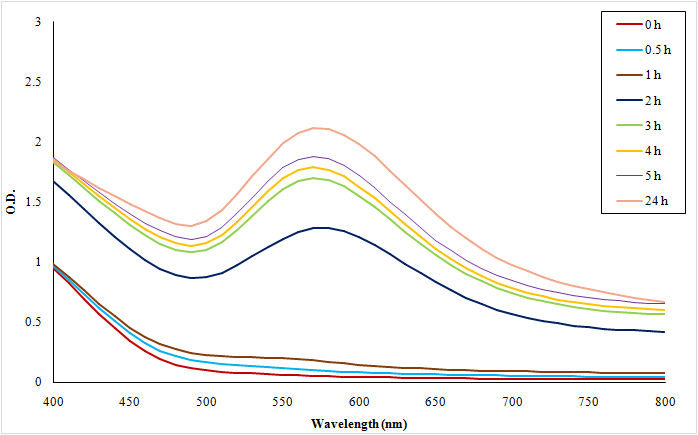


**Figure S23.** UV-visible spectra recorded as a function of reaction time for AuNPs synthesis using LFLE with 1 mM HAuCl_4_ at 20 °C.


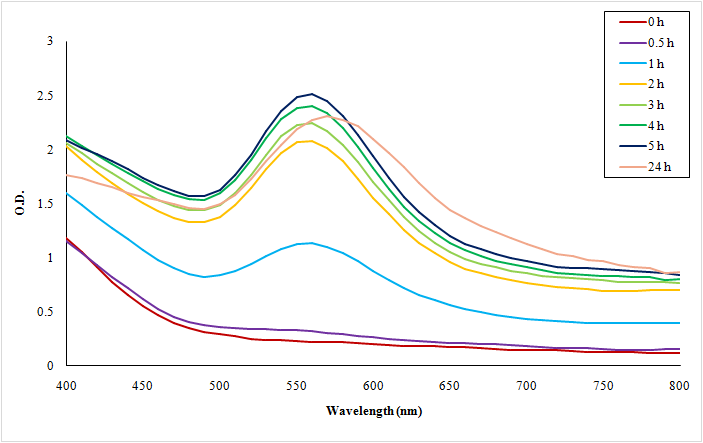


**Figure S24.** UV-visible spectra recorded as a function of reaction time for AuNPs synthesis using LFLE with 1 mM HAuCl_4_ at 30 °C.


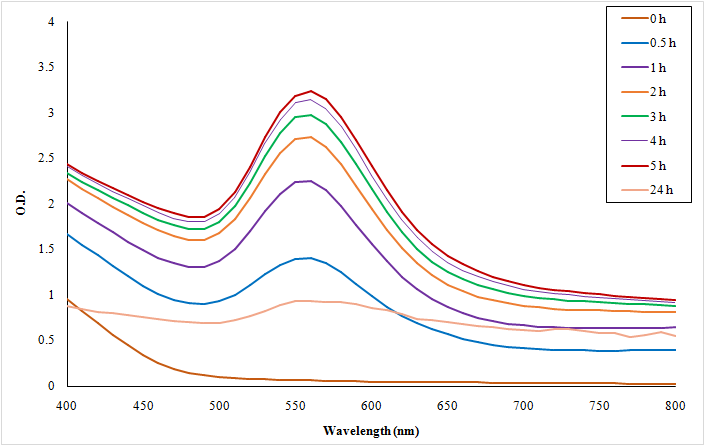


**Figure S25.** UV-visible spectra recorded as a function of reaction time for AuNPs synthesis using LFLE with 1 mM HAuCl_4_ at 40 °C.


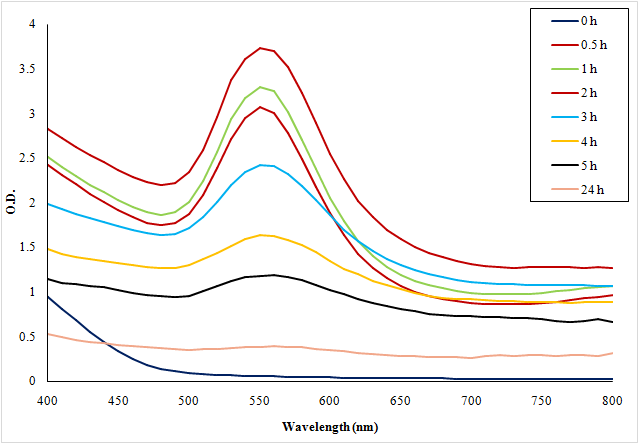


**Figure S26.** UV-visible spectra recorded as a function of reaction time for AuNPs synthesis using LFLE with 1 mM HAuCl_4_ at 50 °C.


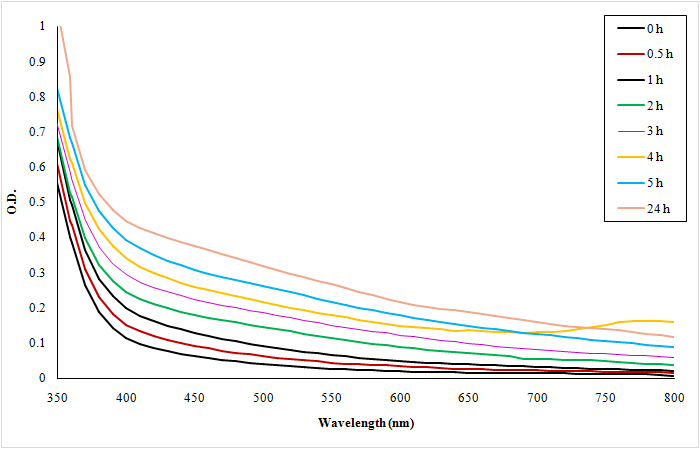


**Figure S27.** UV-visible spectra recorded as a function of reaction time for AgNPs synthesis with 5 mM AgNO_3_ using 1% LFLE.


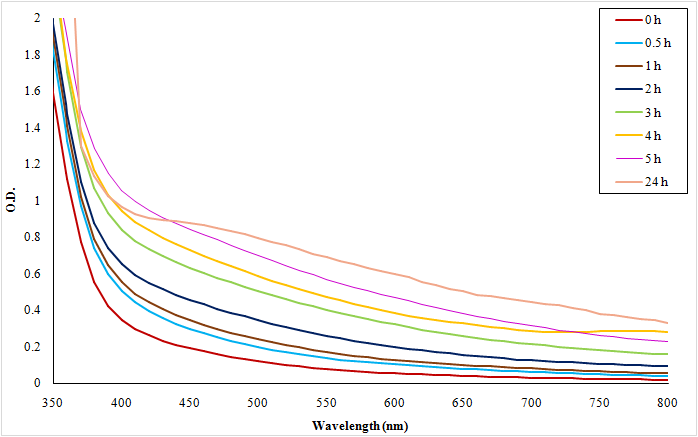


**Figure S28.** UV-visible spectra recorded as a function of reaction time for AgNPs synthesis with 5 mM AgNO_3_ using 3% LFLE.


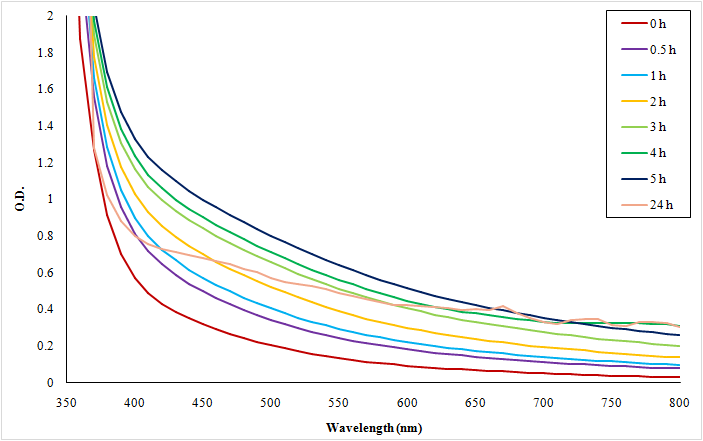


**Figure S29.** UV-visible spectra recorded as a function of reaction time for AgNPs synthesis with 5 mM AgNO_3_ using 5% LFLE.


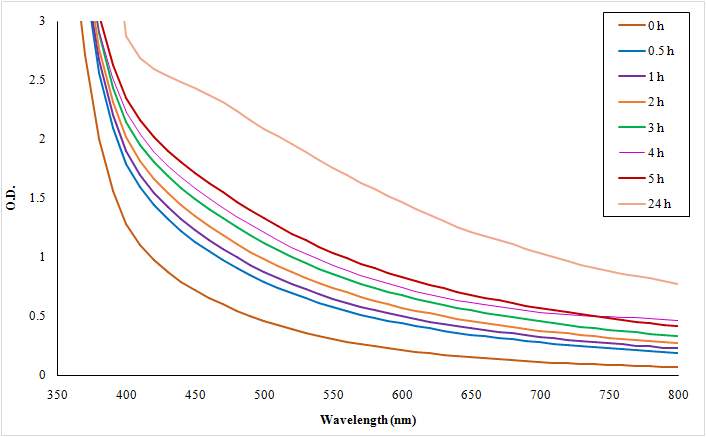


**Figure S30.** UV-visible spectra recorded as a function of reaction time for AgNPs synthesis with 5 mM AgNO_3_ using 10% LFLE.


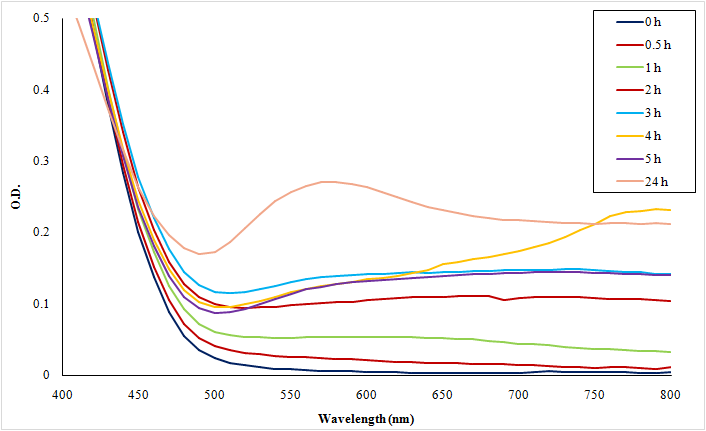


**Figure S31.** UV-visible spectra recorded as a function of reaction time for AuNPs synthesis with 1 mM HAuCl_4_ using 1% LFLE.


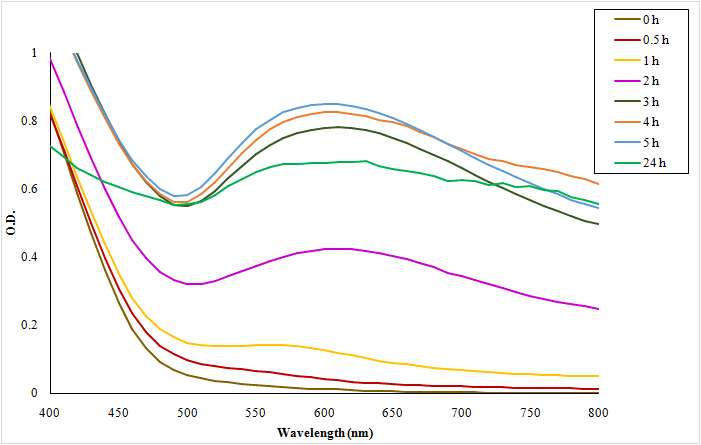


**Figure S32.** UV-visible spectra recorded as a function of reaction time for AuNPs synthesis with 1 mM HAuCl_4_ using 3% LFLE.


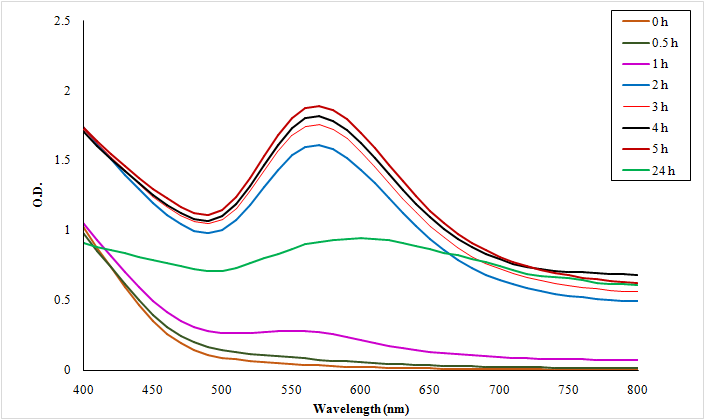


**Figure S33.** UV-visible spectra recorded as a function of reaction time for AuNPs synthesis with 1 mM HAuCl_4_ using 5% LFLE.


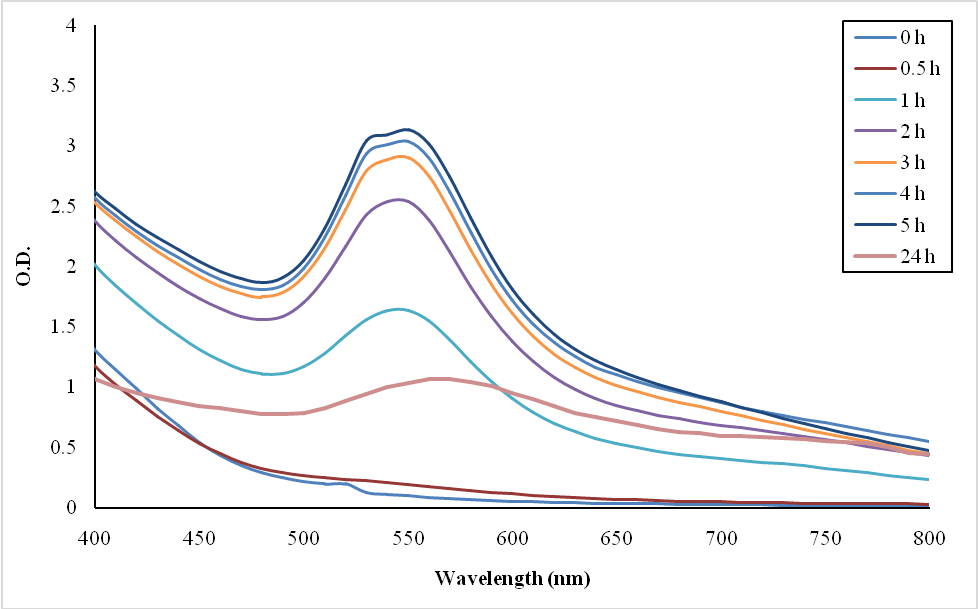


**Figure S34.** UV-visible spectra recorded as a function of reaction time for AuNPs synthesis with 1 mM HAuCl_4_ using 10% LFLE.
